# Insulin resistance increases the risk of conversion to the subcortical small vessel type of dementia^☆^

**DOI:** 10.1016/j.cccb.2026.100561

**Published:** 2026-08-27

**Authors:** Elin Axelsson Andrén, Petronella Kettunen, Anders Wallin, Johan Svensson

**Affiliations:** a Department of Internal Medicine and Clinical Nutrition, Institute of Medicine, Sahlgrenska Academy, University of Gothenburg, Gothenburg, Sweden; b Department of Psychiatry and Neurochemistry, Institute of Neuroscience and Physiology, Sahlgrenska Academy, University of Gothenburg, Mölndal, Sweden; c Department of Neuropsychiatry, Sahlgrenska University Hospital, Region Västra Götaland, Mölndal, Sweden; d Department of Internal Medicine, Skaraborg Central Hospital, Region Västra Götaland, Skövde, Sweden

**Keywords:** Subcortical small vessel type of dementia, Alzheimer’s disease, Mild cognitive impairment, Vascular cognitive impairment, Insulin resistance, HOMA-IR, Cardiovascular risk factors

## Abstract

**Background:**

Insulin resistance (IR) has been associated with cognitive impairment and white matter hyperintensities (WMHs) on magnetic resonance imaging (MRI) in population-based studies. However, in the clinical setting, the impact of IR on the risk of the subcortical small vessel type of dementia (SSVD) is unknown.

**Objective:**

To investigate whether the homeostatic model assessment of insulin resistance (HOMA-IR) was associated with the risk of SSVD or Alzheimer's disease (AD) in a memory clinic cohort.

**Methods:**

We included 352 patients from the Gothenburg MCI study with subjective or mild cognitive impairment (SCI or MCI) at baseline. SSVD was diagnosed based on clinical symptomatology, including predominant frontal lobe symptoms, and WMH amount on MRI. Cox regression, adjusted for covariates, assessed the associations between baseline HOMA-IR and incident SSVD or AD.

**Results:**

During follow-up (mean 4.1 years), 83 (24%) patients developed dementia (SSVD, n = 28; AD, n = 55). Baseline HOMA-IR (per standard deviation increase) was associated with increased risk of SSVD (adjusted hazard ratio [HR] = 1.41, 95% CI: 1.16–1.73). Patients in the highest HOMA-IR quintile had an almost seven-fold higher risk of SSVD than those in the lowest quintile (adjusted HR = 6.87, 95% CI: 1.09–43.38). These associations remained significant also after excluding patients with diabetes. HOMA-IR was not associated with AD.

**Conclusion:**

IR is a strong predictor of SSVD, but not AD, in patients with SCI or MCI. This suggests that IR contributes to SSVD progression and that HOMA-IR may be a useful tool for risk stratification.

## Highlights

• Higher HOMA-IR levels were associated with an increased risk of progression to subcortical small vessel type of dementia.
• HOMA-IR was significantly associated with the risk of subcortical small vessel type of dementia also after exclusion of patients with diabetes.
• Insulin resistance is a strong predictor of the subcortical small vessel type of dementia, but not of Alzheimer’s disease.
• Lifestyle interventions or medical treatments that can reduce insulin resistance may be useful at the early stage of subcortical small vessel type of dementia.

## 1 Introduction

Insulin resistance (IR) is associated with cognitive impairment. Yet, the clinical relevance of IR across clinical populations remains incompletely understood. Cognitive decline has a spectrum of underlying pathologies from Alzheimer’s disease (AD), the most common cognitive disease with hallmarks amyloid and tau pathology [1], to vascular cognitive impairment (VCI) [2]. VCI includes cognitive impairment due to vascular pathologies ranging from stroke-related cognitive disease to the subcortical small vessel type of dementia (SSVD). In clinical practice, mixed pathologies are common, underscoring the continuum from AD to VCI [2].

The prevalence of SSVD is not clear, but it may account for up to 50% of VCI cases [3]. The progression of SSVD is gradual and driven by arteriolosclerosis, hyaline and fibrinoid vessel wall changes, and interstitial edema [3]. These abnormalities compromise the blood–brain barrier, enabling extravasation of large molecules into the white matter, which promotes neuroinflammation and demyelination [3]. This chronic hypoperfusion and myelin destruction can be visualized as white matter hyperintensities (WMHs) on magnetic resonance imaging (MRI) [4]. The clinical symptoms of SSVD include executive dysfunction, impaired self-regulation, unbalanced mood, memory deficits, and in the later stages, functional disability [5].

Metabolic disturbances may accelerate both neurodegenerative and cerebrovascular pathologies as in population-based studies, type 2 diabetes and the metabolic syndrome have been associated with an elevated risk of both AD and VCI [6,7]. However, midlife IR and related metabolic disturbances have been more strongly associated with the risk of AD compared with such disturbances in late life [[8], [9], [10], [11], [12]]. In accordance with these findings, several studies have found that when AD is clinically manifest, body weight may not be increased and the metabolic imbalances are less pronounced than in midlife [[13], [14], [15], [16]]. Similarly, the risk of VCI is increased by derangements of metabolic risk factors in midlife [17,18], but the cardiometabolic abnormalities are, to a higher degree than in AD, still present in clinically manifest VCI [4,[19], [20], [21], [22]]. Previous studies have included stroke-related VCI and mixtures of underlying etiologies [17,18,20,22], but very few studies have assessed metabolic aberrations in study populations only comprising SSVD patients [19,23].

IR participates in the development of conditions such as diabetes, obesity, cardiovascular disease, chronic kidney disease, and non-alcoholic fatty liver disease [24]. A practical and validated marker of peripheral IR is the homeostatic model assessment of insulin resistance (HOMA-IR) [25], calculated from fasting blood glucose and fasting insulin levels. In both large population-based cohorts and smaller community- or hospital-based studies, in which the amount of WMHs on MRI has been used as a proxy of SSVD, elevated HOMA-IR or impairments of other measures of glucose homeostasis have been associated with cognitive impairment [26,27], and increased burden of WMHs in some [[28], [29], [30], [31], [32]], but not all [33], of the studies. However, few of these studies have concomitantly included assessment of IR, neuroimaging and cognitive outcomes [26]. Furthermore, the association between IR and the risk of clinically defined SSVD has previously not been investigated in patients who have sought help for cognitive decline at a memory clinic.

In summary, the association between IR and the risk of SSVD in a memory clinic sample remains unknown. Therefore, we performed a single-center prospective study of well-defined patients with subjective cognitive impairment (SCI) or objective mild cognitive impairment (MCI). In this memory clinic population, we examined whether baseline HOMA-IR was associated with the subsequent risk of conversion to SSVD or AD. We hypothesized that impaired IR would be associated with an increased risk of SSVD in patients with SCI or MCI at baseline.

## 2 Materials and methods

### 2.1 Patients and setting

The patients were recruited from the Gothenburg MCI Study, which is a prospective, single-center study conducted at the Memory Clinic, Sahlgrenska University Hospital, Mölndal, Sweden [34]. Comprehensive evaluations, which included lumbar puncture and MRI, were performed at baseline and at biennial follow-ups to assess the etiology and severity of the cognitive impairment. Inclusion criteria were age 50 – 79 years, Mini-Mental State Examination (MMSE) score > 18, self- or informant-reported cognitive decline lasting ≥ 6 months, and in the present study also having a diagnosis of SCI or MCI at baseline. Exclusion criteria were conditions potentially affecting cognition, such as severe somatic illnesses (e.g., subdural hemorrhage, brain tumor, hypothyroidism, encephalitis, or unstable cardiovascular diseases such as angina pectoris or myocardial infarction) and psychiatric disorders (e.g., major depressive disorder, schizophrenia, substance abuse, and delirium). In the present study, additional exclusion criteria were lack of data on fasting insulin and glucose (to determine HOMA-IR) and CSF biomarkers. Finally, we also excluded patients converting to dementias other than SSVD or AD.

At baseline and at follow-up, the patients were classified using the Global Deterioration Scale (GDS) [35]. GDS 1 indicates no cognitive decline, GDS 2 suggests SCI, GDS 3 is equivalent to MCI, and GDS 4 corresponds to mild dementia. The classification into GDS groups was based on medical history, checklists, and cognitive symptom assessment tools, including Stepwise Comparative Status Analysis (STEP) variables 13–20 [36]; I-FLEX, a short version of the Executive Interview (EXIT) [37]; MMSE [38] and Clinical Dementia Rating (CDR) [39]. In the present study, participants classified as GDS 2 or 3 at baseline were followed longitudinally until they converted to dementia (GDS 4) or to a maximum of 6 years.

GDS 4 was defined as STEP > 1, I-FLEX > 3, CDR > 1.0, and MMSE ≤ 25, although a consensus decision among the specialized physicians at the outpatient ward was required to determine the final GDS score. In patients classified as GDS 4, when setting the specific diagnoses, the specialized physicians had access to clinical symptomatology and estimated WMHs on MRI based on the Fazekas scale [40], but remained blinded to neuropsychological test results and cerebrospinal fluid (CSF) biomarker data.

SSVD was diagnosed using the Erkinjuntti criteria [41]. Specifically, for an SSVD diagnosis, MRI-detected WMHs (mild, moderate, or severe per the Fazekas scale [40]) and predominant frontal lobe symptoms were required. When WMHs were mild, SSVD was diagnosed only in the absence of prominent parietotemporal lobe symptoms (e.g., apraxia, aphasia, or agnosia). This classification also aligns with the concept of subcortical ischemic vascular dementia, which was used in the Vascular Impairment of Cognition Classification Consensus Study (VICCCS) [2]. In addition, patients who progressed to a clinical SSVD diagnosis were excluded if they were positive for the CSF biomarker criteria for AD presented below. AD was diagnosed according to the NINCDS-ADRDA criteria [42]. In addition, in the present study, a biological AD diagnosis required that the patients met CSF biomarker criteria [43] at the time of conversion (amyloid-β_42_ [Aβ_42_] < 530 ng/L, total tau [t-tau] > 350 ng/L, and phosphorylated tau_181_ [p-tau_181_] > 59 ng/L) [44].

Patients who, after conversion to dementia, fulfilled the clinical criteria for both AD and SSVD initially received a clinical diagnosis of mixed dementia (n = 31) [34]. Then, after employment of the CSF biomarker criteria for AD, they were reclassified as AD if they fulfilled the biomarker criteria for AD (n = 20). In contrast, if they did not meet the CSF biomarker criteria for AD, they were classified as SSVD (n = 11).

Fifteen patients who converted to other (non-AD/non-SSVD) dementias were excluded (primary progressive aphasia, n = 1; Lewy body dementia, n = 2; frontotemporal dementia, n = 1; unspecified dementia, n = 11). Patients who did not convert to dementia during follow-up were classified as stable SCI/MCI.

**Fig. 1:**
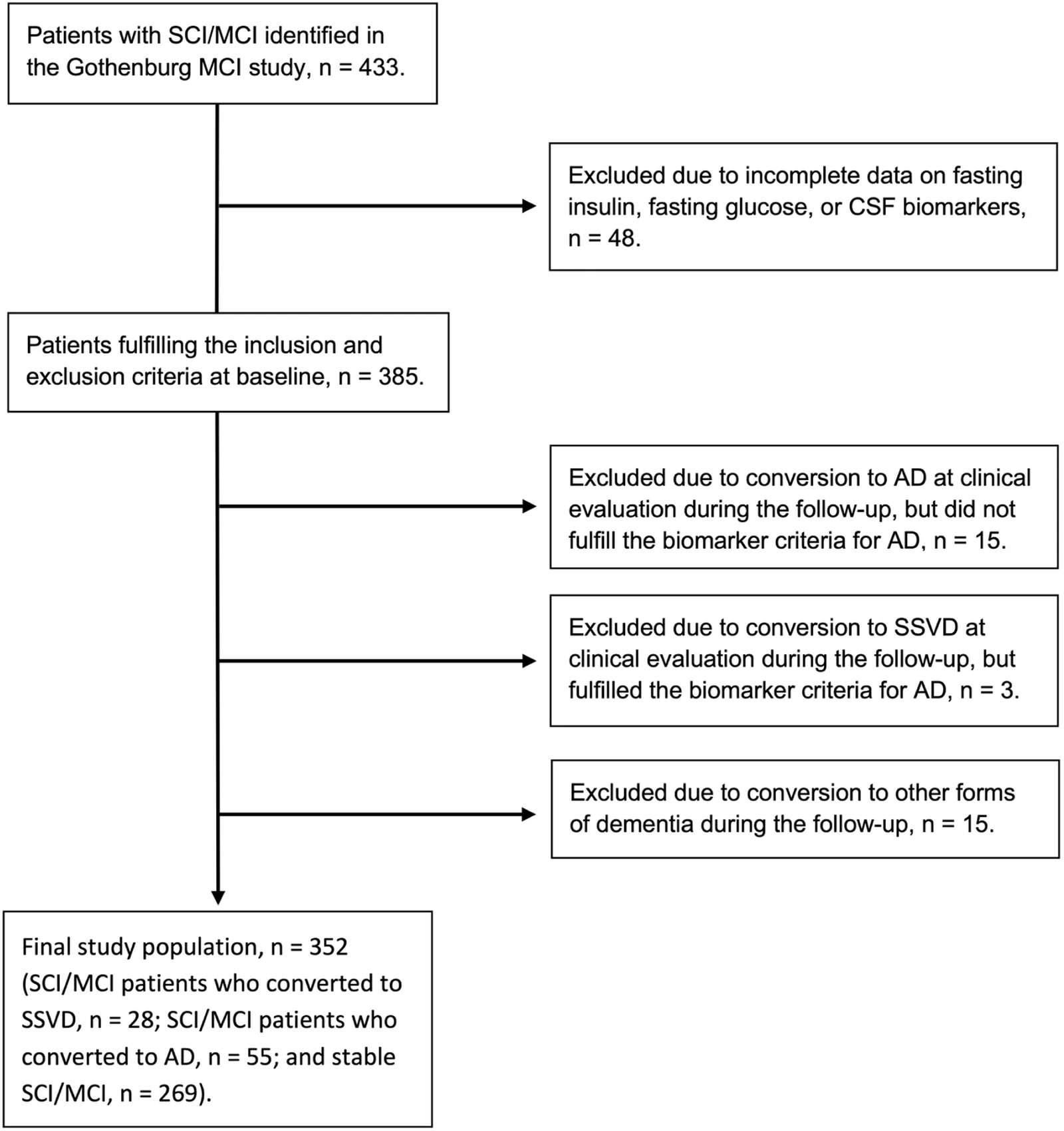
Flow chart of the inclusion and exclusion of patients in the present study. Of the 433 patients with SCI or MCI identified in the Gothenburg MCI study, 48 patients were excluded due to incomplete data in terms of fasting insulin, fasting glucose, or CSF biomarker data. Among the remaining 385 patients, additional exclusions were made for conversion to other (non-AD/non-SSVD) dementias (n = 15), clinical conversion to AD without fulfilling CSF AD biomarker criteria (n = 15), and clinical conversion to SSVD while fulfilling AD biomarker criteria (n = 3). The final sample (n = 352) included 28 patients with SCI/MCI at baseline who converted to SSVD, 55 patients with SCI/MCI at baseline who converted to AD, and 269 patients with stable SCI or MCI during follow-up. SCI, subjective cognitive impairment; MCI, mild cognitive impairment; n, numbers; CSF cerebrospinal fluid; AD, Alzheimer's disease; SSVD, subcortical small vessel type of dementia.

In summary, we initially identified 433 patients with SCI or MCI at baseline (Fig. 1). Of these patients, 385 had complete data on fasting insulin and glucose (to determine HOMA-IR) and CSF biomarkers. Then, we excluded 15 patients who converted to a clinical diagnosis of AD but did not fulfill the CSF AD biomarker criteria, 3 patients who progressed to a clinical diagnosis of SSVD but met the biomarker criteria for AD, and 15 patients who converted to other forms of dementia. Therefore, our final sample encompassed 352 patients (28 of these patients converted from SCI/MCI to SSVD during follow-up, 55 patients progressed from SCI/MCI to AD, and 269 patients had stable SCI/MCI).

### 2.2 Cardiovascular risk factors

At the baseline visit, a memory clinic physician recorded the presence of hypertension, diabetes mellitus (hereafter diabetes) and smoking status (nonsmoker, former smoker, or present smoker). Hypertension was defined as the use of antihypertensive treatment or blood pressure measurements (in repeated assessments) ≥ 140/90 mmHg, or ≥ 130/80 mmHg for diabetic individuals [45]. Diabetes was defined according to the World Health Organization (WHO) 2006/2011 criteria [46], which includes fasting plasma glucose ≥ 7.0 mmol/L (126 mg/dL), a two-hour plasma glucose ≥ 11.1 mmol/L (200 mg/dL) during an oral glucose tolerance test, or treatment with oral antidiabetics and/or insulin. Prediabetes was defined as fasting plasma glucose ≥ 6.1 but < 7 mmol/L, or HbA1c ≥ 42 but < 48 mmol/mol, or a two-hour plasma glucose ≥ 7.8 but < 11.1 mmol/L during an oral glucose tolerance test. Body mass index (BMI) was calculated using the formula: weight in kilograms / height in meters squared, and HOMA-IR using the formula: fasting insulin * fasting glucose / 22.5.

### 2.3 Neuropsychological tests

In addition to the tests used for GDS classification, we used the Trail Making Test A (TMT-A) and B (TMT-B) to evaluate visual scanning and complex attention [34].

### 2.4 Blood and CSF collection

Blood samples were collected between 8:00 a.m. and 10:00 a.m. after an overnight fast, and CSF samples were obtained between 8:00 a.m. and 12:00 pm. to minimize diurnal variation. Lumbar puncture was performed in all patients at baseline and at follow-up (when a dementia diagnosis was set). The lumbar puncture was performed at the L3-L5 intervertebral space and CSF was collected in polypropylene tubes. The first tube was discarded to prevent blood contamination. A total of 20 mL of CSF was collected, centrifuged at 2000 × g for 10 min at room temperature, and stored in cryotubes at −80 °C for later analysis [34].

### 2.5 Biochemical methods

The personnel were blinded to clinical information. Plasma glucose, serum insulin and serum lipid measurements were performed using standardized methods (accredited according to international standard ISO/IEC 15,189) at the Clinical Chemistry Laboratory, Sahlgrenska University Hospital, Gothenburg, Sweden. CSF analysis was conducted at the Clinical Neurochemistry Laboratory, Sahlgrenska University Hospital, Mölndal, Sweden. AD biomarkers (Aβ_42_, t-tau, and p-tau_181_) were quantified using INNOTEST® ELISA assays (Fujirebio, Ghent, Belgium). Two internal control samples (pooled CSF aliquots) were analyzed in each run to ensure assay quality. The intra- and inter-assay coefficients of variation were under 10%. *APOE* genotyping was performed using mini sequencing [34].

### 2.6 Statistical analyses

The statistical analyses were conducted using SPSS version 30 (IBM Corp., Armonk, NY). Descriptive data are presented as means with standard deviations (SD). HOMA-IR was logarithmically transformed in the analyses as the distribution was right-skewed. Between-group differences were assessed using analysis of variance (ANOVA) followed by Tukey’s post hoc test for continuous variables and using chi-square tests for categorical variables.

Cox proportional hazards regression models were employed to calculate hazard ratios (HRs) and 95% confidence intervals (CIs) for the associations between baseline HOMA-IR and the risk of progression to dementia (SSVD or AD). In these analyses, HOMA-IR was analyzed as a standardized continuous variable [per SD increase]. In addition, we performed Cox proportional hazards regression analyses to calculate the risk of SSVD or AD per quintile increase in HOMA-IR as well as whether the risk of SSVD differed between individual quintiles using the lowest (most beneficial) HOMA-IR quintile as reference. For the Cox proportional hazards regression analyses, unadjusted (crude) associations between HOMA-IR and dementia risk are presented, or an adjusted model, which included adjustment for age, sex, education, diabetes at baseline, hypertension, smoking, BMI, total cholesterol, and the *APOE ε4* allele. Finally, Kaplan–Meier survival curves were used to estimate the risk of incident dementia in the lowest (most beneficial) HOMA-IR quintile (Q1), the three middle quintiles (Q2-Q4), and Q5. Significance was obtained if the two-tailed *p*-value was ≤ 0.05.

## 3 Results

### 3.1 Clinical characteristics at baseline

**Fig. 2:**
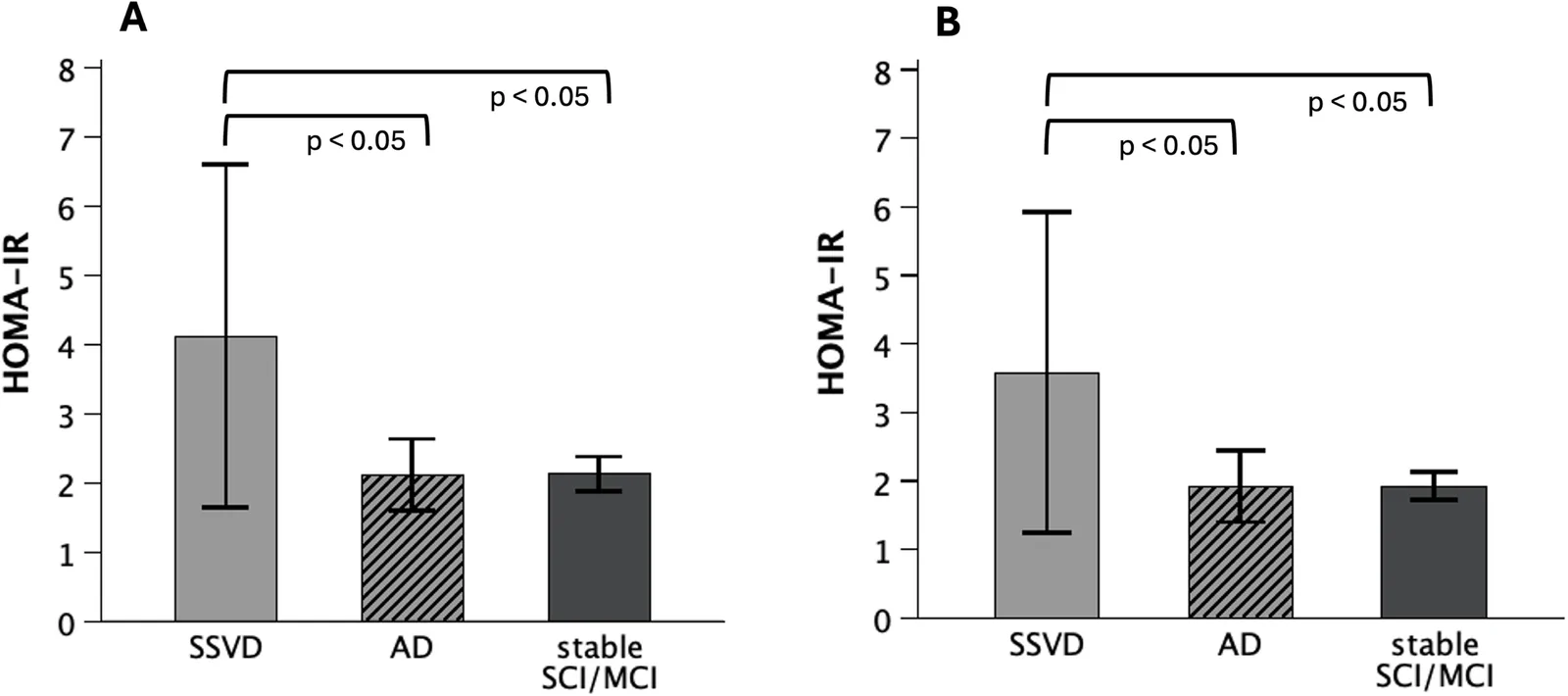
Baseline levels of HOMA-IR in the total study population (n = 352) and after exclusion of diabetic patients (n = 334). **(A)** In the total study population, baseline HOMA-IR was higher in patients who later progressed to SSVD (n = 28) compared with patients who later converted to AD (n = 55) and stable SCI/MCI (n = 269). **(B)** Also, in the non-diabetic patients, patients who later converted to SSVD (n = 26) had higher baseline HOMA-IR than patients who later progressed to AD (n = 50) and stable SCI/MCI (n = 258). Values are given as means (height of bars) and 95% CI (error bars). Between-group differences were calculated using ANOVA followed by Tukey’s post hoc test. HOMA-IR, Homeostatic model assessment of insulin resistance; CI, confidence interval; SSVD, subcortical small vessel type of dementia; AD, Alzheimer's disease; SCI, subjective cognitive impairment; MCI, mild cognitive impairment; ANOVA, analysis of variance.

Patients with SCI or MCI (n = 352) were followed for a maximum of 6 years (mean follow-up time 4.1 years, range was 2 to 6 years). The patients that converted to SSVD during the follow-up visits (n = 28) exhibited elevated baseline HOMA-IR levels compared with patients who converted to AD (n = 55, *p* < 0.05) and stable SCI/MCI patients (n = 269, *p* < 0.05) (Table 1 and Fig. 2A). Furthermore, in a subanalysis in which patients with diabetes at baseline (n = 18) were excluded (remaining patients, n = 334), baseline HOMA-IR was still elevated in patients later converting to SSVD compared with patients who progressed to AD (*p* < 0.05) and patients with stable SCI or MCI (*p* < 0.05) (Fig. 2B).

**Table 1:** Baseline characteristics in patients with SCI or MCI who later converted to SSVD or AD or had stable SCI/MCI.

Variables at baseline	SSVD	AD	Stable SCI/MCI	P-value between groups
	(n = 28)	(n = 55)	(n = 269)	
HOMA-IR	**4.1 (6.4) ^a, c^**	2.1 (2.1)	2.1 (2.1)	**0.01**
Age (years)	**68.8 (7.1) ^c^**	**67.5 (6.8) ^c^**	63.8 (7.3)	**<0.001**
Men / Women (n, %) Education Ed	17 / 11 (60.7 / 39.3)	23 / 32 (41.8 / 58.2)	116 / 153 (43.1 / 56.9)	0.19
Education (years)	**11.1 (3.7) ^c^**	11.8 (3.7)	12.9 (3.5)	**0.01**
Prediabetes yes/no (n, %)	3 / 25 (10.7 / 89.3)	3 / 52 (5.5 / 94.5)	31 / 238 (11.5 / 88.5)	0.83
Diabetes yes/no (n, %)	2 / 26 (7.1 / 92.9)	5 / 50 (9.1 / 90.9)	11 / 258 (4.1 / 95.9)	0.27
Hypertension yes/no (n, %)	20/8 (71.4 / 28.6)	29/26 (52.7 / 47.3)	137/132 (50.9 / 49.1)	0.12
Smoking yes / no / former (n,%)	4 / 13 / 11 (14.3 / 46.4 / 39.3)	4 / 38 / 13 (7.3 / 69.1 / 23.6)	20 / 140 / 109 (7.4 / 52.0 / 40.5)	0.10
BMI (kg/m^2^)	25.4 (3.2)	23.9 (3.4)	25.1 (3.5)	0.05
Total cholesterol (mmol/L)	5.7 (0.8)	5.9 (1.1)	5.6 (1.0)	0.17
MMSE score	**28.0 (2.1) ^c^**	**27.5 (1.7) ^b^**	28.7 (1.2)	**<0.001**
TMT-A time (seconds)	**54.0 (18.2) ^b^**	**54.4 (21.3) ^b^**	39.0 (13.8)	**<0.001**
TMT-B time (seconds)	**162.0 (74.1) ^b^**	**145.4 (64.7) ^b^**	90.7 (39.0)	**<0.001**
*APOE ε4* alleles, 0 / I / II (n, %)	**14 / 10 / 2 (53.8 / 38.5 / 7.7) ^a^**	**12 / 25 / 15 (23.1 / 48.1 / 28.8) ^b^**	140 / 90 / 17 (56.7 / 36.4 / 6.9)	**<0.001**

Values are presented as means (SD) if not otherwise stated. All patients had SCI or MCI at baseline. Group differences were analyzed using analysis of variance (ANOVA) with Tukey’s post hoc test for continuous variables and using chi-square tests for categorical variables. *APOE ε4* genotyping was unavailable in 27 patients. Significant differences are marked with bold text. **^a^** p < 0.05 vs AD; ^b^ p < 0.001 vs stable SCI/MCI; ^c^ p < 0.05 vs stable SCI/MCI.

MCI, mild cognitive impairment; SSVD, subcortical small vessel type of dementia; AD, Alzheimer's disease; SCI, subjective cognitive impairment; HOMA-IR, Homeostatic model assessment of insulin resistance; BMI, body mass index; MMSE, Mini Mental State Examination; TMT, Trail Making Test; *APOE;* apolipoprotein E.

The baseline characteristics of the study population (n = 352) is given in Table 1. The SCI/MCI patients in the impending SSVD and AD groups were older than those in the stable SCI/MCI group (both *p* < 0.05), but there was no difference in sex distribution. Years of education were lower in the group that later progressed to SSVD compared with stable SCI/MCI (*p* < 0.05). The baseline prevalence of prediabetes, diabetes, hypertension, and smoking status as well BMI and total cholesterol did not differ significantly between groups (Table 1). Scores of MMSE, TMT-A, and TMT-B were impaired in the SCI/MCI patients converting to SSVD or AD compared with the stable SCI/MCI group. *APOE ε4* allele frequency was higher in SCI/MCI patients who later converted to AD compared with those who progressed to SSVD (*p* < 0.05) and stable SCI/MCI (*p* < 0.001).

At baseline, 18 patients had diabetes. Of these, 11 patients received oral antidiabetic treatment, two were on insulin therapy, and five did not receive medication. During follow-up, three patients with oral antidiabetic treatment also received insulin therapy, one untreated patient were initiated on oral antidiabetic treatment, and one untreated patient started insulin therapy.

Nine patients received a diagnosis of diabetes during the follow-up. One of these patients later converted to SSVD, two patients subsequently progressed to AD, and six patients had stable SCI/MCI. Four of these patients started oral antidiabetic therapy during the follow-up.

### 3.2 CSF biomarkers at baseline and at conversion to dementia

CSF biomarker levels at baseline and at conversion to SSVD or AD are presented in Table 2. As expected, at baseline as well as at conversion to dementia, CSF AD biomarker levels differed between patients with AD and the other study groups, consistent with the applied biomarker-supported diagnostic criteria.

**Table 2:** CSF biomarker concentrations at baseline and at the time of dementia diagnosis in patients with SCI or MCI at baseline who later converted to SSVD or AD or had stable SCI/MCI.

Variable	SSVD	AD	Stable SCI/MCI	*P*-value between groups
	(n = 28)	(n = 55)	(n = 269)	
Lumbar puncture at baseline				
CSF Aβ_42_ (ng/L)	**570.7 (180.9) ^a, c^**	**391.6 (91.1) ^b^**	670.6 (226.3)	**<0.001**
CSF t-tau (ng/L)	**360.6 (183.0) ^a^**	**784.6 (403.1) ^b^**	327.9 (176.5)	**<0.001**
CSF p-tau_181_ (ng/L)	**51.8 (18.4) ^a^**	**96.4 (37.4) ^b^**	54.3 (27.5)	**<0.001**
Lumbar puncture at the time of dementia diagnosis				
CSF Aβ_42_ (ng/L)	**554.5 (219.8) ^a^**	343.4 (83.3)	N/A	**<0.001**
CSF t-tau (ng/L)	**353.5 (143.1) ^a^**	772.1 (356.2)	N/A	**<0.001**
CSF p-tau_181_ (ng/L)	**53.7 (16.5) ^a^**	102.4 (41.5)	N/A	**<0.001**

Values are presented as means (SD). All patients had SCI or MCI at baseline. Group differences were analyzed using analysis of variance (ANOVA) followed by Tukey’s post hoc test. Significant differences are marked with bold text. **^a^** p < 0.001 vs AD; ^b^ p < 0.001 vs stable SCI/MCI; ^c^ p < 0.05 vs stable SCI/MCI.

CSF, cerebrospinal fluid; SCI, subjective cognitive impairment; MCI, mild cognitive impairment; N/A, not applicable, SSVD, subcortical small vessel type of dementia; AD, Alzheimer's disease; Aβ_42_, amyloid-β_42_; t-tau, total tau; p-tau_181_, phosphorylated tau_181_.

### 3.3 HOMA-IR (per SD increase) and the risk of conversion to dementia

We performed Cox proportional hazards regression analyses in which HOMA-IR at baseline was used as a standardized continuous variable (Table 3). These analyses showed that baseline HOMA-IR (per SD increase) was significantly associated with increased risk of conversion to SSVD both in the crude model (HR = 1.35, 95% CI: 1.14–1.60, *p* < 0.001) and in the adjusted model (HR = 1.41, 95% CI: 1.16–1.73, *p* < 0.001). In contrast, there was no association between baseline HOMA-IR (per SD increase) and the risk of AD (Table 3).

**Table 3:** Hazard ratios for the risk of conversion to SSVD or AD per SD or quintile increase in HOMA-IR in the entire study population and in patients without diabetes at baseline.

Total study population (n = 352)				
	Incident SSVD (n = 28)		Incident AD (n = 55)	
	HR (95% CI)	P-value	HR (95% CI)	P-value
Per SD increase				
HOMA-IR, crude	**1.35 (1.14 – 1.60)**	**<0.001**	0.93 (0.67 – 1.28)	0.63
HOMA-IR, adjusted*	**1.41 (1.16 – 1.73)**	**<0.001**	0.85 (0.58 – 1.28)	0.43
Per quintile increase				
HOMA-IR Q1–5, crude	**1.46 (1.10 – 1.93)**	**0.009**	1.04 (0.86 – 1.26)	0.67
HOMA-IR Q1–5, adjusted*	**1.44 (1.02 – 2.05)**	**<0.05**	1.07 (0.84 – 1.36)	0.58

Patients without diabetes at baseline (n = 334)				
	Incident SSVD (n = 26)		Incident AD (n = 50)	
Per SD increase				
HOMA-IR, crude	**1.35 (1.13 – 1.64)**	**0.001**	0.92 (0.62 – 1.38)	0.69
HOMA-IR, adjusted⁎⁎	**1.42 (1.14 – 1.76)**	**0.002**	0.91 (0.62 – 1.32)	0.60
Per quintile increase				
HOMA-IR Q1–5, crude	**1.50 (1.11 – 2.01)**	**0.008**	1.00 (0.82 – 1.22)	0.99
HOMA-IR Q1–5 adjusted⁎⁎	**1.46 (1.02 – 2.09)**	**<0.05**	1.06 (0.84 – 1.35)	0.62

Hazard ratios (HRs), 95% confidence intervals (CIs), and *p*-values for the risk of conversion to SSVD or AD were calculated using Cox proportional hazards regression analyses. Significant associations are marked with bold text.

SSVD, subcortical small vessel type of dementia; AD, Alzheimer's disease; HR, hazard ratio; CI, confidence interval; HOMA-IR, Homeostatic model assessment of insulin resistance; Q, quintile; BMI, body mass index; *APOE;* apolipoprotein E.

⁎ Adjustment for age, sex, education, diabetes at baseline, hypertension, smoking, BMI, total cholesterol, and *APOE* ε4 allele.

⁎⁎ Adjustment for age, sex, education, hypertension, smoking, BMI, total cholesterol, and *APOE* ε4 allele.

### 3.4 Analyses of quintiles of baseline HOMA-IR in relation to SSVD risk

HRs and 95% CIs for the risk of SSVD and AD per quintile increase in baseline HOMA-IR are presented in Table 3. Also in these analyses, baseline HOMA-IR was associated with an elevated risk of SSVD (per quintile increase; crude: HR = 1.46, 95% CI: 1.10–1.93, *p* < 0.01; adjusted: HR = 1.44, 95% CI: 1.02–2.05, *p* < 0.05). Furthermore, there was no association between baseline HOMA-IR (per quintile increase) and the risk of AD (Table 3).

**Fig. 3:**
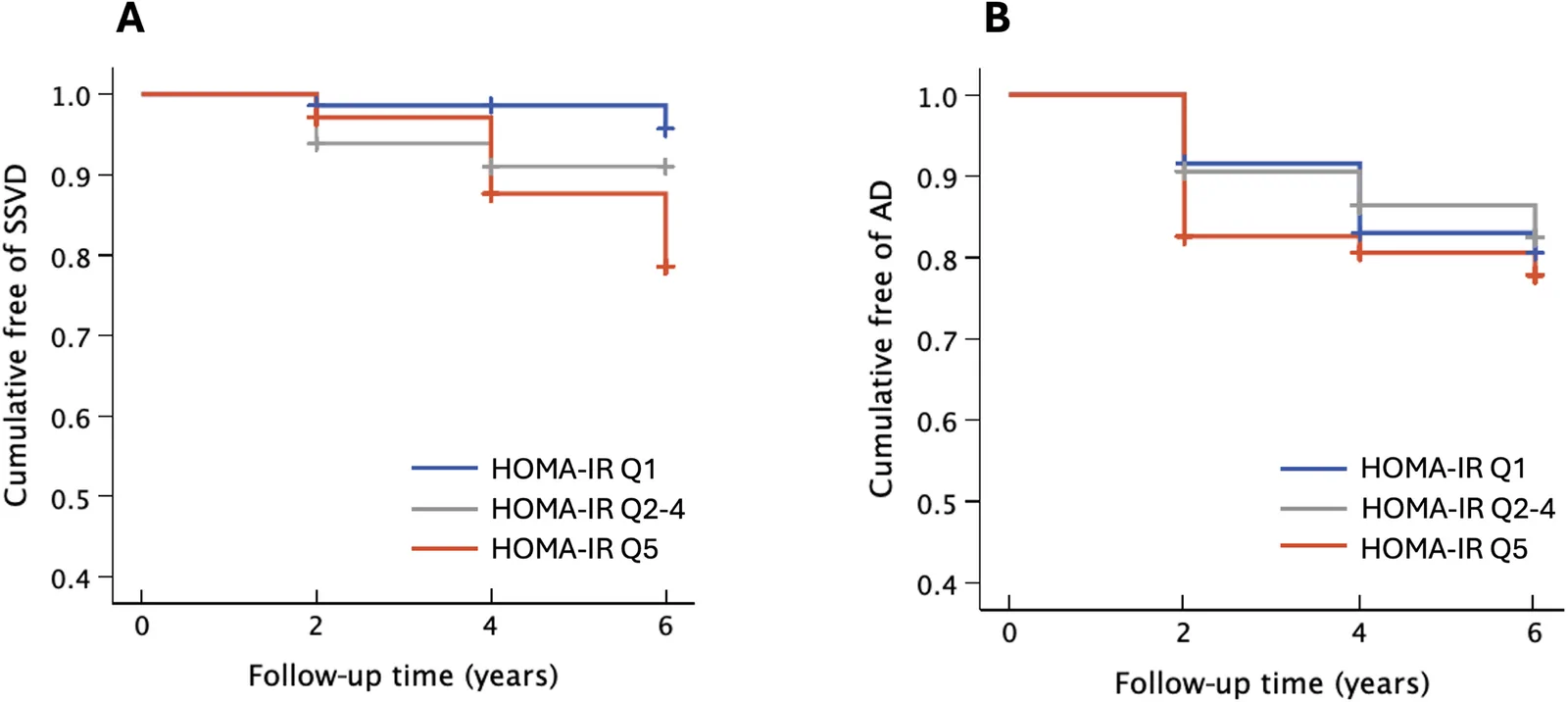
The highest quintile of baseline HOMA-IR was associated with increased risk of conversion to SSVD, but not with the risk of AD, in patients with SCI or MCI at baseline. Kaplan-Meier estimators are presented for **(A)** the risk of conversion to SSVD in quintiles of baseline HOMA-IR (Q5 vs. Q1; log-rank test, p < 0.05) and **(B)** the risk of progression to AD in quintiles of baseline HOMA-IR (Q5 vs. Q1; log-rank test, p = 0.46). The risk of SSVD or AD was similar in Q2 - Q4 of baseline HOMA-IR compared with Q1. Blue, HOMA-IR Q1; grey, HOMA-IR Q2 - Q4; red, HOMA-IR Q5. HOMA-IR, Homeostatic model assessment of insulin resistance; SSVD, subcortical small vessel type of dementia; SCI, subjective cognitive impairment; MCI, mild cognitive impairment; AD, Alzheimer's disease; Q, quintile.

Next, we analyzed whether the risk of SSVD differed between individual quintiles of baseline HOMA-IR using the lowest (the most beneficial) HOMA-IR quintile (Q1) as reference (Table 4). In these analyses, the risk of SSVD was statistically similar in Q2, Q3, and Q4 of HOMA-IR compared with Q1, whereas Q5 of HOMA-IR was associated with a significantly elevated risk of conversion to SSVD (Q5 vs. Q1: crude: HR = 4.89, 95% CI: 1.06–22.66, *p* < 0.05; adjusted: HR = 6.87, 95% CI: 1.09 – 43.38, *p* < 0.05) (Table 4). Finally, cumulative survival curves confirmed that Q5 of baseline HOMA-IR was associated with increased SSVD risk compared with the lowest quintile (Q5 vs. Q1; log-rank test, *p* < 0.05, Fig. 3A), whereas there was no association with the risk of AD (Fig. 3B).

**Table 4:** Hazard ratios for the risk of conversion to SSVD in individual quintiles of HOMA-IR in the entire study population and in non-diabetic patients.

	Total study population		Patients without diabetes at baseline	
	(n = 352)		(n = 334)	
	Incident SSVD (n = 28)		Incident SSVD (n = 26)	
	Crude model		Crude model	
Quintile	HR (95% CI)	*p*-value	HR (95% CI)	*p*-value
Q1	Referent	-	Referent	-
Q2	1.50 (0.25 – 8.99)	0.66	1.50 (0.25 – 8.99)	0.66
Q3	3.07 (0.62 – 15.22)	0.17	3.07 (0.62 – 15.22)	0.17
Q4	4.18 (0.89 – 19.70)	0.07	3.78 (0.78 – 18.22)	0.10
Q5	**4.89 (1.06 – 22.66)**	**<0.05**	**5.41 (1.15 – 25.46)**	**<0.05**

	Adjusted model*		Adjusted model⁎⁎	
Quintile	HR (95% CI)	*p*-value	HR (95% CI)	*p*-value
Q1	Referent	-	Referent	-
Q2	0.60 (0.05 – 6.83)	0.68	0.60 (0.05 – 6.83)	0.68
Q3	1.98 (0.33 – 11.78)	0.45	1.98 (0.33 – 11.78)	0.45
Q4	2.26 (0.39 – 13.29)	0.37	2.20 (0.38 – 12.87)	0.38
Q5	**6.87 (1.09 – 43.38)**	**<0.05**	**6.84 (1.01 – 46.19)**	**<0.05**

Hazard ratios (HRs), 95% CIs, and p-values for the risk of conversion to SSVD were calculated using Cox proportional hazards regression analyses. Significant associations are marked with bold text.

SSVD, subcortical small vessel type of dementia; HOMA-IR, Homeostatic model assessment of insulin resistance; HR, hazard ratio; CI, confidence interval; Q, quintile; BMI, body mass index; *APOE*; apolipoprotein E.

⁎ Adjustment for sex, education, diabetes at baseline, hypertension, smoking, BMI, total cholesterol, and *APOE* ε4 allele.

⁎⁎ Adjustment for age, sex, education, hypertension, smoking, BMI, total cholesterol, and *APOE* ε4 allele.

### 3.5 HOMA-IR and the risk of conversion to dementia in patients without diabetes

We performed subanalyses in which patients with diabetes at baseline (n = 18) were excluded. In the remaining patients (n = 334), higher baseline HOMA-IR was still associated with increased risk of SSVD (per SD increase: adjusted model: HR = 1.42, 95% CI: 1.14 – 1.76, *p* = 0.002; per quintile increase: adjusted model: HR = 1.46, 95% CI: 1.02 – 2.09, *p* < 0.05) (Table 3). Likewise, the association between the highest HOMA-IR quintile and increased risk of SSVD remained significant in patients without diabetes at baseline (Q5 vs. Q1: adjusted model: HR = 6.84, 95% CI: 1.01 – 46.19, *p* < 0.05) (Table 4). Finally, cumulative survival curves confirmed that the highest quintile of baseline HOMA-IR was associated with increased SSVD risk in patients without diabetes at baseline (Q5 vs. Q1; log-rank test, p < 0.05; not shown).

We also performed subanalyses in which we excluded patients with diabetes at baseline (n = 18) as well as patients who developed diabetes during the follow-up (n = 9). In the remaining patients (n = 325), higher baseline HOMA-IR (per SD increase) was still associated with increased risk of SSVD (crude: HR = 1.37, 95% CI: 1.13 – 1.65, *p* = 0.001; adjusted: HR = 1.43, 95% CI: 1.14 – 1.78, *p* = 0.002, not shown). Furthermore, HOMA-IR (per quintile increase) was still associated with increased risk of SSVD in the crude model (HR = 1.47, 95% CI: 1.09 – 1.98, *p* < 0.05), but this association marginally lost statistical significance after adjustment for covariates (adjusted model: HR = 1.42, 95% CI: 0.994 – 2.04, *p* = 0.05; not shown). However, the increased risk of SSVD in the highest HOMA-IR quintile remained significant (Q5 vs. Q1: crude: HR = 4.97, 95% CI: 1.03 – 23.9, *p* < 0.05; adjusted: HR = 7.45, 95% CI: 1.03 – 53.99, *p* < 0.05) (not shown). As expected, in this selection of patients, HOMA-IR was not associated with the risk of AD (data not shown).

### 3.6 HOMA-IR and the risk of dementia in patients without diabetes and prediabetes

In further subanalyses, we excluded patients with diabetes at baseline (n = 18), patients who developed diabetes during follow-up (n = 9), and patients with prediabetes at baseline (n = 37). In the remaining patients with SCI/MCI at baseline (n = 294), higher baseline HOMA-IR (per SD increase) was still associated with increased risk of SSVD (crude: HR = 1.53, 95% CI: 1.07 – 2.19, *p* < 0.05; adjusted: HR = 1.59, 95% CI: 1.01 – 2.51, *p* < 0.05; not shown). HOMA-IR (per quintile increase) was associated with increased risk of SSVD in the crude model (HR = 1.44, 95% CI: 1.05 – 1.98, *p* < 0.05; not shown), but not in the adjusted model (HR = 1.27, 95% CI: 0.86 – 1.86, *p* = 0.23). Furthermore, the risk of conversion to SSVD was no longer significant between the highest and the lowest quintile of HOMA-IR (crude: HR = 4.69, 95% CI: 0.91 – 24.19, *p* = 0.07; adjusted: HR = 3.31, 95% CI: 0.29 – 37.83, *p* = 0.34; not shown). There was no association between HOMA-IR and the risk of AD in this group of patients (data not shown).

## 4 Discussion

In this single-center prospective study of patients with SCI or MCI at baseline, we investigated whether baseline HOMA-IR was associated with the risk of conversion to dementia in a memory clinic population. We found, both in the total study population, and in subanalyses of patients without diabetes, that higher baseline HOMA-IR levels were significantly associated with greater risk of progression to clinically manifest SSVD also after adjustment for covariates. Furthermore, after adjustment for covariates, patients in the highest HOMA-IR quintile had an almost sevenfold increased risk of SSVD compared with those in the lowest quintile. In contrast, there was no association between baseline HOMA-IR and the risk of progression to AD.

The central finding of the present study is that in patients with SCI or MCI at baseline, IR estimated by HOMA-IR was markedly associated with increased risk of progression to SSVD. Previously, impaired glucose homeostasis has been associated with increased risk of VCI, but the majority of the studies have been performed in stroke-related VCI or in VCI populations with various underlying etiologies [21,22]. In population-based [[31], [32], [33]] or smaller community- or hospital-based [[28], [29], [30],^47^] studies, in which the amount of WMHs on MRI has frequently been used to estimate the degree of subcortical small-vessel disease, elevated HOMA-IR or impairments of other measures of glucose homeostasis have been associated with increased WMH burden in most [[28], [29], [30], [31], [32],^47^], but not all [33], of the studies. In the largest study performed so far (34,789 adults without prior stroke or dementia in the UK Biobank cohort), lower estimated glucose disposal rate (eGDR), a marker of IR, was linearly associated with greater amount of WMHs [31]. However, in the Leukoaraiosis and Disability study (LADIS; 639 elderly individuals), the associations between diabetes and impaired baseline cognition [48] or cognitive decline over 3 years [49] were independent of the amount of WMHs. In contrast, a hospital-based study including 329 patients with signs of small vessel disease (including WMHs) on MRI showed that impaired HOMA-IR was associated with worse performance on the Montreal Cognitive Assessment (MoCA) test [26]. Therefore, overall, the relations between IR/glucose homeostasis, WMH burden, and cognitive decline have not been clarified. Moreover, in a memory clinic population, the association between IR and SSVD risk is unknown. We therefore extend the previous knowledge by showing that in patients seeking help for cognitive decline, impaired baseline HOMA-IR was associated with increased risk of conversion from SCI/MCI to manifest SSVD defined by typical clinical symptomatology and presence of WMHs on MRI).

In our total study population, which included 18 patients with diabetes at baseline and 9 patients who received a diagnosis of diabetes during the follow-up, higher HOMA-IR was associated with increased risk of SSVD. In most but not all [50] earlier studies, diabetes has been associated with increased WMH burden [30,49,51,52], cognitive decline [48,49], and increased dementia risk [6]. In addition, we previously demonstrated an increased prevalence of diabetes in clinically manifest SSVD compared with AD dementia and healthy controls [19]. In diabetes, the transportation of glucose and insulin across the blood-brain barrier is altered [53], which affects the regional metabolism and the microcirculation in the brain [54]. Prolonged chronic hyperglycemia may then further alter membrane permeability and decrease the regional blood flow, resulting in permanent cell damage [55]. Therefore, we investigated whether our results were dependent on an uneven distribution of diabetes. In subanalyses in which we excluded patients with diabetes at baseline (n = 18), all associations between elevated baseline HOMA-IR and increased SSVD risk were still statistically significant. Furthermore, in additional subanalyses, we excluded not only patients with diabetes at baseline, but also patients who were diagnosed with diabetes during the follow-up. In the latter analyses, all associations between HOMA-IR and SSVD risk remained significant except for the association between HOMA-IR (per quintile increase) and increased SSVD risk, which marginally lost statistical significance (*p* = 0.05). Therefore, overall, the association between higher HOMA-IR and increased SSVD risk was not to any major extent dependent of the distribution of diabetes.

We also performed subanalyses in which we excluded all patients with diabetes (n = 27) as well as patients with prediabetes at baseline (n = 37). In the remaining patients, some of the associations between higher HOMA-IR and increased SSVD risk lost statistical significance, such as the increased risk of SSVD in the highest HOMA-IR quintile compared with the lowest quintile. Therefore, we cannot exclude the possibility that not only IR, but also the distribution of prediabetes, contributed to the association between HOMA-IR and increased SSVD risk. However, reduced statistical power due to the limited number of patients in these subgroup analyses could also have influenced the results, and the importance of prediabetes for the risk of SSVD needs to be investigated in more detail in future studies.

In the present study, there was no significant association between baseline HOMA-IR and the risk of conversion to AD. Although impaired cardiovascular risk factors have been associated with increased risk of AD [6,7], metabolic disturbances (including IR) in midlife have been more markedly associated with AD risk in previous studies compared with late life measurements of cardiometabolic factors [[8], [9], [10], [11]]. Furthermore, when clinical symptoms of AD are apparent, most of the metabolic abnormalities seen in midlife have already been reversed [[13], [14], [15], [16]]. Therefore, overall, the lack of association between HOMA-IR and the risk of AD in our study is in line with the results of population-based studies showing that late life measurements of cardiometabolic risk factors are not, or only weakly, associated with the risk of AD [11,13]. This may in turn suggest that intervention strategies, such as lifestyle intervention or medical treatment, are probably more effective in pre-symptomatic stages of AD. Furthermore, in the brain, there may be an insulin resistant state with reduced signaling through the insulin receptor in neurons, glia, and vascular cells [56]. As insulin normally supports neuronal survival and synaptic function, IR in the brain may lead to neuronal degeneration due to loss of trophic signals [56,57]. Furthermore, IR in the brain can result in inflammatory and vascular disturbances including endothelial dysfunction, impaired vasoreactivity, and aberrant inflammatory signaling [26,56,58]. This could eventually result in chronic cerebral hypoperfusion, blood–brain barrier disruption, and white matter injury [26,55], and it is therefore tempting to speculate that IR in the brain may cause or accelerate the progression of SSVD. However, the association between brain IR and SSVD development remains insufficiently understood and needs to be explored in more detail in future studies.

Experimental studies [59] and postmortem human studies [57] have shown that the AD brain is insulin resistant. Furthermore, insulin and Aβ are both degraded by insulin degrading enzyme. Additionally, impaired neuronal insulin signaling participates in synaptic failure, Aβ accumulation and tau hyperphosphorylation, which are key features of AD pathology [12,56,57,60,61]. However, in our study, there was no association between HOMA-IR and the risk of progression to AD, which is in line with the previous finding that diabetes is related to cerebral infarction but not to AD pathology in older persons [62]. One possible explanation for this lack of association in our study and the earlier study [62] could be that aberrations in brain insulin regulation are confined to manifest AD dementia and thus are not present to any major extent in the SCI or MCI stage of AD. Alternatively, it is possible that the changes in insulin regulation seen in the AD brain are not reflected in peripheral measures of insulin action such as HOMA-IR.

In the present study, IR was associated with increased risk of conversion from SCI/MCI to clinically manifest SSVD, which suggests that systemic metabolic dysfunction is involved in SSVD progression. Measuring HOMA-IR may therefore help to identify individuals with SCI or MCI who are at risk of converting to SSVD. Furthermore, there are some indications that lifestyle interventions or medical treatment can improve cognition in diabetic patients [[63], [64], [65], [66]]. Although there is scarce clinical evidence, it could be speculated that therapeutic strategies aimed at improving glucose homeostasis or enhancing central insulin signaling could be of benefit also in non-diabetic patients in the SCI or MCI stage of SSVD. Such therapies may include diet and exercise intervention or medical treatments such as intranasal insulin [63], metformin [66], incretin-based therapies such as Glucagon-Like Peptide-1 (GLP-1) receptor agonists [64], or through indirect pathways (for example Sodium–Glucose Cotransporter 2 [SGLT-2] inhibitors) [65]. However, further studies are clearly needed to study these issues in detail.

A major strength of the present study is the single center memory clinic setting. All our patients were well-characterized and had symptoms of cognitive impairment, which contrasts to previous population-based studies in which the presence of WMHs on MRI have been used as a proxy of SSVD [[31], [32], [33]]. Also, the clinical setting makes the results translatable to patients seeking help for early cognitive deterioration. The limitations include that only a relatively small number of the patients converted to SSVD in our study, which may have decreased the statistical power**.** Another limitation is that HOMA-IR as well as the proportion of patients who had prediabetes was assessed only at baseline as a complete evaluation of glucose homeostasis was only performed at the start of the study. Furthermore, the association that we observed between elevated HOMA-IR and increased risk of conversion to SSVD was more marked than those previously seen between IR or diabetes type 2 and the amount of WMHs (as proxy for SSVD) or cognitive decline in several population-based studies [6,28,33,48]. Consequently, our results need to be interpreted with caution, and firm conclusions regarding the association between IR and the risk of SSVD cannot be drawn based on the present study alone. Moreover, mixed dementia (combined AD and SSVD) was not studied as a separate diagnostic group. Finally, only patients of European (Swedish) ancestry were included, which limits the generalizability and our findings may therefore not be applicable to populations with different genetic, environmental, or sociocultural backgrounds.

## 5 Conclusions

In this prospective memory clinic study of patients with SCI or MCI, we found that IR, estimated by HOMA-IR, was associated with increased risk of conversion to SSVD independent of the presence of diabetes. In contrast, HOMA-IR was not associated with the risk of AD. Further studies are needed to characterize the risk factor panorama in SSVD compared with AD in more detail. Moreover, future studies need to investigate whether lifestyle interventions or medical treatments can be of value at the SCI or MCI stage of SSVD when the patients seek help at a memory clinic.

## Funding

This work was supported by the Swedish state under the agreement between the Swedish government and the county councils, the ALF-agreement (ALFGBG-965,744 [Johan Svensson], ALFGBG-720,661 [Anders Wallin] and ALFGBG-724,331 / ALFGBG-1006,983 [Petronella Kettunen]). Elin Axelsson Andrén is supported by Stiftelsen Psykiatriska Forskningsfonden, Sweden. Petronella Kettunen is supported by the following Swedish foundations: Alzheimerfonden; Gunvor och Josef Anérs stiftelse; Greta och Einar Askers stiftelse; Magnus Bergvalls Stiftelse; Anna-Lisa och Bror Björnssons stiftelse; Demensfonden; Stiftelsen för Gamla Tjänarinnor; Maud & Birger Gustavssons Stiftelser; Hedlunds stiftelse; Kungliga Vetenskaps- och Vitterhetssamhället i Göteborg; Axel Linders Stiftelse; Wilhelm & Martina Lundgrens Vetenskapsfond; Stiftelsen Längmanska kulturfonden; Agneta Prytz-Folkes och Gösta Folkes stiftelse; Stiftelsen Psykiatriska Forskningsfonden; Stiftelsen Handlanden Hjalmar Svenssons Forskningsfond; Syskonen Svenssons Fond För Medicinsk Forskning; Gun och Bertil Stohnes Stiftelse; The Wenner-Gren Foundations; Stiftelsen familjen Nils Winbergs fond and Åhlénstiftelsen. Anders Wallin is supported by Konung Gustaf V:s och Drottning Victorias Frimurarestiftelse, Sweden. Johan Svensson is supported by Demensfonden, Sweden.

## Ethical considerations

This study was approved by the regional ethical committee (diary number: L091– 99 and T479– 11) and the Swedish Ethical Review Authority (diary number: 2020–06,733). The study was conducted in accordance with the Declaration of Helsinki. Written informed consent was obtained from all patients.

## CRediT authorship contribution statement

**Elin Axelsson Andrén:** Writing – original draft, Methodology, Formal analysis, Conceptualization. **Petronella Kettunen:** Writing – review & editing, Resources, Investigation. **Anders Wallin:** Writing – review & editing, Resources, Investigation. **Johan Svensson:** Writing – original draft, Supervision, Methodology, Formal analysis, Conceptualization.

## Declaration of competing interest

The authors declare the following financial interests/personal relationships which may be considered as potential competing interests:

Elin Axelsson Andren reports financial support was provided by Stiftelsen psykiatriska forskningsfonden. Anders Wallin reports financial support was provided by the ALF agreement. Johan Svensson reports financial support was provided by the ALF agreement. Petronella Kettunen reports financial support was provided by the ALF agreement. Petronella Kettunen reports financial support was provided by Alzheimersfonden. Petronella Kettunen reports financial support was provided by Gunvor och Josef Anérs stiftelse. Petronella Kettunen reports financial support was provided by Greta och Einar Askers stiftelse. Petronella Kettunen reports financial support was provided by Magnus Bergvalls Stiftelse. Petronella Kettunen reports financial support was provided by Anna-Lisa och Bror Björnssons stiftelse. Petronella Kettunen reports financial support was provided by Demensfonden. Petronella Kettunen reports financial support was provided by Stiftelsen för Gamla Tjänarinnor. Petronella Kettunen reports financial support was provided by Maud & Birger Gustavssons Stiftelser. Petronella Kettunen reports financial support was provided by Hedlunds stiftelse. Petronella Kettunen reports financial support was provided by Kungliga Vetenskaps- och Vitterhetssamhället i Göteborg. Petronella Kettunen reports financial support was provided by Axel Linders Stiftelse. Petronella Kettunen reports financial support was provided by Wilhelm & Martina Lundgrens Vetenskapsfond. Petronella Kettunen reports financial support was provided by Stiftelsen Längmanska kulturfonden. Petronella Kettunen reports financial support was provided by Agneta Prytz-Folkes och Gösta Folkes stiftelse. Petronella Kettunen reports financial support was provided by Stiftelsen Psykiatriska Forskningsfonden. Petronella Kettunen reports financial support was provided by Stiftelsen Handlanden Hjalmar Svenssons Forskningsfond. Petronella Kettunen reports financial support was provided by Syskonen Svenssons Fond För Medicinsk Forskning. Petronella Kettunen reports financial support was provided by Gun och Bertil Stohnes Stiftelse. Petronella Kettunen reports financial support was provided by The Wenner-Gren Foundations. Petronella Kettunen reports financial support was provided by Stiftelsen familjen Nils Winbergs fond. Petronella Kettunen reports financial support was provided by Ahlenstiftelsen. Anders Wallin reports financial support was provided by King Gustaf V and Queen Victoria’s Masonic Foundation. Johan Svensson reports financial support was provided by Demensfonden. Anders Wallin is Editor-in-chief of Cerebral Circulation - Cognition & Behavior (CCCB). If there are other authors, they declare that they have no known competing financial interests or personal relationships that could have appeared to influence the work reported in this paper.

## Data Availability

The data supporting the findings of this study are available upon request from the corresponding author. Due to privacy and ethical restrictions, the data are not publicly accessible.
